# PepMANDIS: A Peptide Selection Tool for Designing Function-Based Targeted Proteomic Assays in Complex Microbial Systems

**DOI:** 10.3389/fchem.2021.722087

**Published:** 2021-08-16

**Authors:** Matej Medvecky, Manolis Mandalakis

**Affiliations:** ^1^ Institute of Biodiversity, Animal Health and Comparative Medicine, University of Glasgow, Glasgow, United Kingdom; ^2^ Central European Institute of Technology, University of Veterinary Sciences Brno, Brno, Czech Republic; ^3^ Veterinary Research Institute, Brno, Czech Republic; ^4^ Institute of Marine Biology, Biotechnology and Aquaculture, Hellenic Centre for Marine Research, Heraklion, Greece

**Keywords:** targeted metaproteomics, in silico peptide selection, LC-MS/MS, microbial functioning, environmental proteomics, microbial ecology, bioinformatics tool

## Abstract

The majority of studies focusing on microbial functioning in various environments are based on DNA or RNA sequencing techniques that have inherent limitations and usually provide a distorted picture about the functional status of the studied system. Untargeted proteomics is better suited for that purpose, but it suffers from low efficiency when applied in complex consortia. In practice, the scanning capabilities of the currently employed LC-MS/MS systems provide limited coverage of key-acting proteins, hardly allowing a semiquantitative assessment of the most abundant ones from most prevalent species. When particular biological processes of high importance are under investigation, the analysis of specific proteins using targeted proteomics is a more appropriate strategy as it offers superior sensitivity and comes with the added benefits of increased throughput, dynamic range and selectivity. However, the development of targeted assays requires *a priori* knowledge regarding the optimal peptides to be screened for each protein of interest. In complex, multi-species systems, a specific biochemical process may be driven by a large number of homologous proteins having considerable differences in their amino acid sequence, complicating LC-MS/MS detection. To overcome the complexity of such systems, we have developed an automated pipeline that interrogates UniProt database or user-created protein datasets (e.g. from metagenomic studies) to gather homolog proteins with a defined functional role and extract respective peptide sequences, while it computes several protein/peptide properties and relevant statistics to deduce a small list of the most representative, process-specific and LC-MS/MS-amenable peptides for the microbial enzymatic activity of interest.

## Importance

Targeted proteomics is a powerful analytical tool for sensitive detection and precise quantification of selected proteins in biological samples. This is of particular importance in life sciences and the field of microbiology in particular, as it allows tracing specific microbial processes at the molecular level and elucidating the functional role of isolated microorganisms. However, in complex systems hosting mixed microbial communities, a specific biochemical process may be catalysed by numerous homologous proteins with divergent amino acid sequences, which complicates the application of LC-MS/MS-based targeted proteomic methods for the detection of key-acting proteins. Currently, there is lack of software for the assistance of targeted metaproteomic studies in complex natural habitats. Here, we introduce and describe PepMANDIS, a novel tool intending to assist analytical chemists in the development of function-based targeted proteomic assays for systems harboring mixed microbial communities. PepMANDIS calculates various properties of trypsin-generated peptides from long lists of homologous proteins and provides guidance for shortlisting target peptides that will combine functional specificity, extensive cross-species coverage and LC-MS/MS detectability. We believe that this new approach will help to gain targeted insights about microbial functioning in natural systems and enable quantitative monitoring of key biochemical processes.

## Introduction

Microorganisms are literally everywhere in the environment and are the key drivers of countless processes, such as organic matter remineralization, pollution remediation and global biogeochemical cycling of elements/chemicals. Most of the studies concerning microbial environmental functioning are based on DNA or RNA sequencing (e.g. 16S rRNA gene metagenomics), shotgun metagenomics (i.e. sequencing of total DNA from environmental extracts) or metatranscriptomics (i.e. sequencing of total RNA content) (Huang et al., 2016). However, these techniques have several limitations. The 16S rRNA metagenomics analysis targets a single ribosomal gene and thus provides information only about the identity of microorganisms and the microbial diversity of the system under investigation without revealing any direct information on microbial functioning. The latter is inferred by compiling the functional traits reported in the literature for the identified species. On the other hand, shotgun metagenomics cannot clarify if the identified DNA sequences originate from live or dead/inactive cells and whether the identified genes are actively expressed or not. Metatranscriptomics has been considered as an alternative approach for the elucidation of microbial functioning. However, RNA is labile and prone to degradation, making transcriptomic analysis a challenging task, especially in environmental samples. More importantly, several studies have shown that the levels of mRNA molecules in cells are not always correlated with the levels of encoded proteins (Maier et al., 2009; Vogel and Marcotte, 2012), which are the ultimate functional entities, implying that transcriptomic profiling may provide an unclear picture of microbial functionalities.

Over the last two decades, proteomics made outstanding progress in biomedical sciences and the understanding of biological processes at the molecular level (Cayer et al., 2016; Shi et al., 2016; Hristova and Chan, 2019). At the same time, there has been an increasing interest about the potential applications of this technique in the fields of microbial ecology and environmental chemistry (Schulze, 2005; Sowell et al., 2011; Zorz et al., 2019). The majority of relevant studies were oriented towards the complete cataloguing of proteins in native environments. However, the scanning capabilities of the LC-MS/MS systems currently employed for untargeted proteomics are far from providing exhaustive coverage of complex microbial consortia and they are able to unveil only the most abundant proteins expressed by the most abundant microorganisms. Moreover, a great part of them are house-keeping proteins providing relatively limited biological information about distinct, habitat-related functions (Gouveia et al., 2019). Undoubtedly, protein-based stable isotope probing (protein-SIP) has been a valuable proteomic approach for exploring the functional characteristics of microbial communities through the metabolic incorporation of isotopically labeled substrates (e.g. ^13^C, ^15^N) (von Bergen et al., 2013). There are dozens of such quantitative studies investigating the changes in microbial community structure and function, the assimilation pathways of organic compounds and nutrients, as well as the functional role of individual proteins in microbial processes (Bryson et al., 2016; Taubert et al., 2018). However, protein-SIP relies on the cultivation of sampled microorganisms in controlled laboratory experiments, which hardly maintain the conditions and the microbial composition encountered in natural habitats.

Instead of global proteomic profiling, targeted analysis of specific proteins is a much more appropriate strategy when particular biological processes of high importance are under investigation (Saito et al., 2014; Saito et al., 2015). Typically, triple quadrupole LC-MS/MS systems operating in Selected Reaction Monitoring (SRM) mode are employed for tracking process-related peptides/proteins. This methodology is inherently more sensitive and comes with the added benefits of increased throughput, dynamic range and selectivity. However, the application of targeted proteomics requires *a priori* knowledge of the target peptides and their detection-related characteristics, such as their elution time, ionization efficiency and SRM-transitions. When focusing on a single organism with sequenced genome (e.g. human), the development of efficient SRM assays is relatively easy, as a particular biological function is assigned to specific proteins with unambiguously defined amino acid sequences. Considering the broad genetic diversity of microorganisms living in natural habitats, a specific biochemical process may be driven by a large number of homologous proteins having considerable differences in their amino acid sequence, which would complicate LC-MS/MS detection. Moreover, environmental microbial communities are most likely dominated by unsequenced microorganisms with unknown amino acid sequences.

Up to date, a few bioinformatics tools have been developed for examining peptides in large metaproteomic datasets or multiple peptidomes and classifying them according to their functional and taxonomic attributes, with Unipept by Mesuere et al. (2015) (Gurdeep Singh et al., 2019) being the most popular platform. This is a web application implemented in Ruby and JavaScript, which offers impressive capabilities for interactive data visualization. However, it neither provides extensive flexibility in pinpointing unique peptide sequences among all registered homologous proteins, nor is capable of examining the suitability of selected peptides towards LC-MS/MS detection. Here we present PepMANDIS, a Pythonic solution explicitly developed to facilitate the design of targeted proteomic assays for LC-MS/MS metaproteomic studies. It is an automated pipeline that interrogates UniProt or a user-created protein database to gather homolog proteins with defined functional role and extract respective peptide sequences, while it computes several protein/peptide properties and calculates relevant statistics to deduce a small list of the most representative, process-specific and MS-amenable peptides for a microbial enzymatic activity of interest.

## Methods

### Algorithm Overview

**FIGURE 1:**
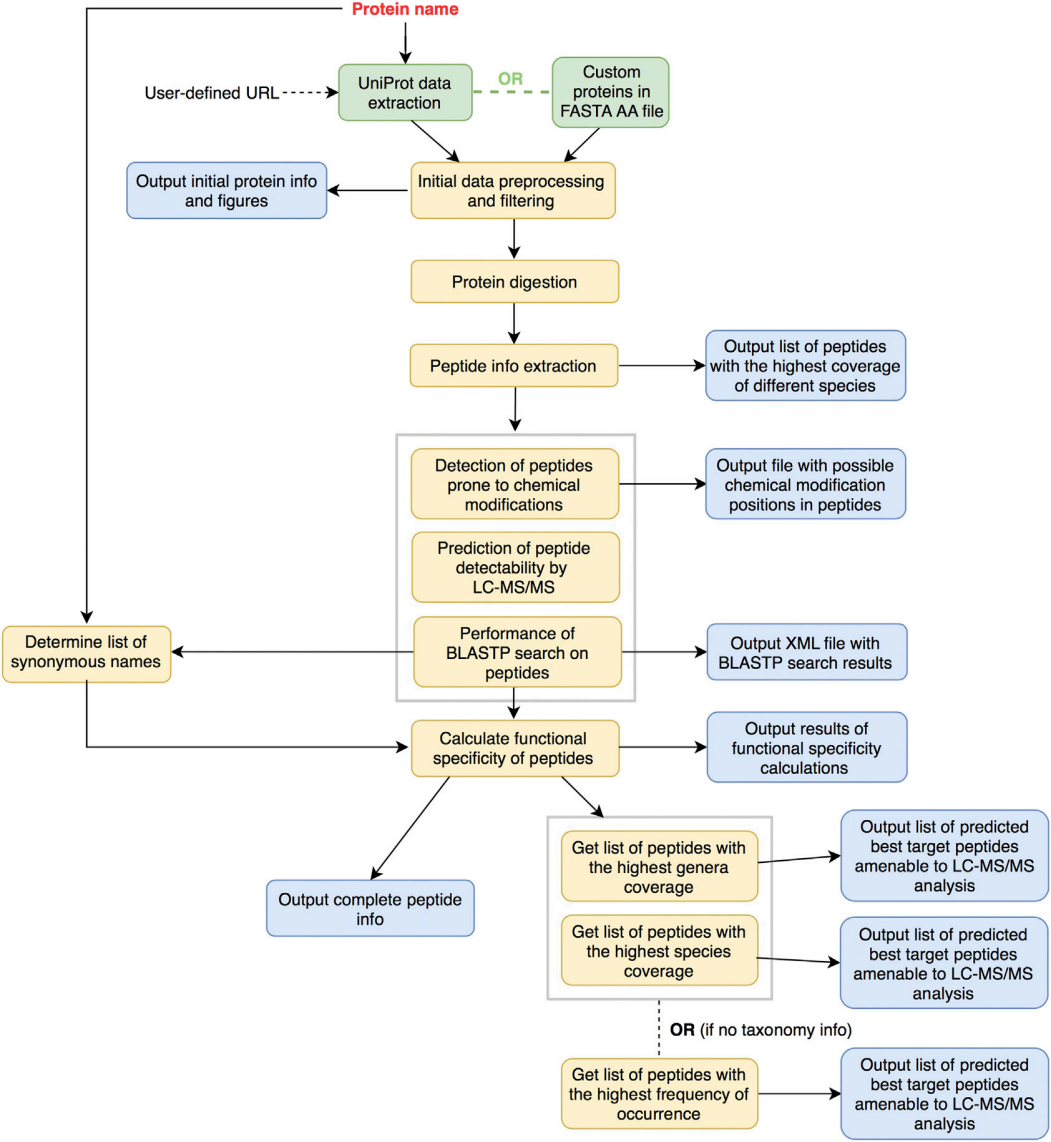
Overview of general peptide prediction workflow as implemented in PepMANDIS. Input sequences are either gathered from UniProt DB or provided as a file in FASTA amino acid format. They are consequently digested proteolytically *in silico* and subjected to the next steps of data processing.

**FIGURE 2:**
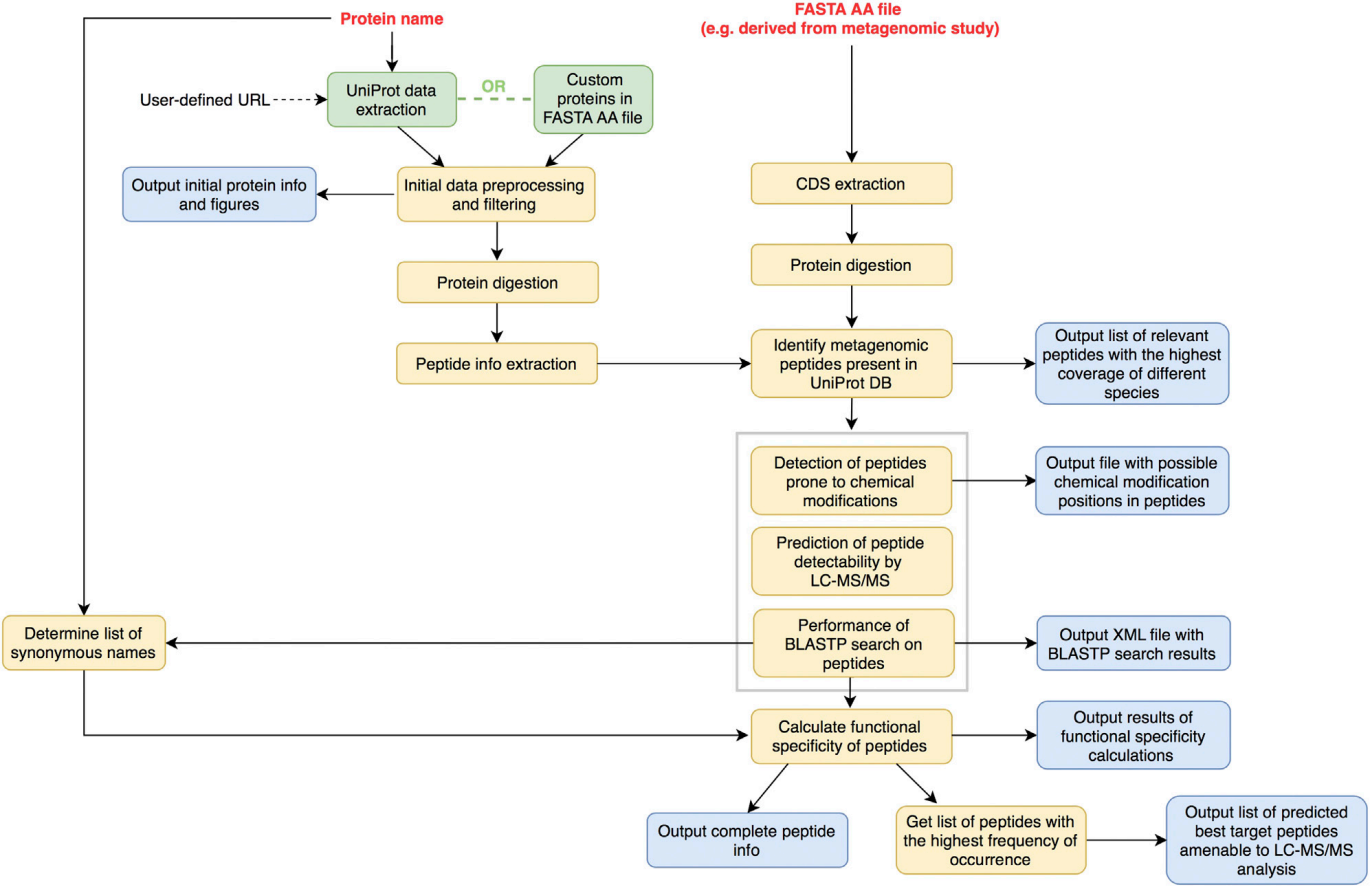
Overview of optional peptide prediction mode workflow. User provides extra input file with protein sequences in FASTA AA format (e.g. derived from metagenomic sequencing). These proteins are digested *in silico* and resulting peptides are compared to those derived from either UniProt DB or other trusted sources of functionally annotated proteins. Exact matches only are then subjected to downstream data processing.

PepMANDIS was written in *Python* v3 and it was tested on several Linux distributions (Fedora, Ubuntu and CentOS), macOS (High Sierra, Big Sur) and Windows (v10). It supports two different peptide prediction modes, the procedures of which are illustrated in Figure 1 and Figure 2. In both modes, the “protein name” is a mandatory option that users have to declare. It is assumed that the users are familiar with the metabolic pathway describing the biological process of interest (e.g. KEGG pathways, https://www.genome.jp/kegg/pathway.html; UM-BBD database, http://eawag-bbd.ethz.ch) and the protein catalyzing a critical step in this pathway has been identified. The name of the protein is thus used to programmatically query UniProt database and track down all relevant entries, and to generate a list of potential protein synonyms reported in BLAST non-redundant protein DB to facilitate peptide specificity calculations in later steps of the pipeline. In the first basic mode, PepMANDIS can download from UniProt DB all the protein sequences classified with the specified protein name, but it also allows the user to set specific taxonomy restrictions (i.e. retrieve sequences from specific groups of microorganisms; by default, PepMANDIS retrieves bacteria-derived sequences only). Moreover, it can accept as input a custom database with pre-selected protein sequences of desired functionality, such as those resulting from an annotated metagenomic assembly file. Indeed, the proteins sequences emanating from metagenomic data of the study system are preferable, because these are more likely to be detectable in the system compared to the non-site-specific proteins listed in UniProt.

Initial input data are gathered and consequently preprocessed (extraction of taxonomy information and protein lengths). Proteins with lengths out of the specified tolerance are filtered out (by default, proteins can vary in size up to 25% from the median length value) and some summary statistics about the proteins are output (e.g. average protein size and protein length standard deviation before and after initial filtering; protein counts per phyla/genera/species). Afterwards, proteins are digested *in silico*, critical features regarding the peptides (e.g. frequency of occurrence among proteins, coverage of microbial species) are extracted and calculated, and the list of peptides having the highest contribution to the coverage of microbial species are outputted. This list is further scrutinized to identify peptides prone to chemical modifications (i.e. those being more challenging to quantify by LC–MS/MS) and estimate their LC-MS/MS detectability and functional specificity (i.e. whether a peptide is present in proteins with the desired function only or not). In the final stage, comprehensive information about the peptides resulting from the *in silico* digestion of input proteins and having a specified length (by default, from 8 amino acid residues (AA) up to 25 AA) is outputted together with two lists containing best target peptide predictions (i.e. peptides with the highest frequency of occurrence across proteins and passing all the filtering options, such as size, functional specificity and MS detectability thresholds and number of possible chemical modifications). The first list encompasses peptides that collectively cover as many as possible of the microbial genera known to encode the target peptide/protein, while the second list represents the highest coverage in terms of microbial species). If the input is a user-defined database of protein sequences, a single list with the best predicted target peptides is only outputted due to the absence of taxonomy information.

In the second mode, the user provides a FASTA file with the AA sequences of proteins assigned to the functional class of choice (e.g. derived from metagenomic data). Protein sequences are extracted from this file, digested *in silico* and the resulting peptide sequences are compared to those of homologous proteins retrieved either from UniProt DB or other trusted sources of functionally annotated proteins. Only the peptide sequences showing an exact match are selected and subjected to the next steps of data processing. In this way, the user is assured that the best target peptides predictions from metagenomic data represent peptides previously reported in databases.

### Input and Output

As a mandatory input, the user specifies protein name which is then used for downloading the initial protein dataset programmatically from the UniProt DB using Selenium libraries (http://www.seleniumhq.org). The initial dataset includes information such as protein ID and names, taxonomy (organism and phylum) and AA sequence along with its length. This UniProt DB search can be restricted to a particular taxonomy specified by the user as an input parameter (by default, this option is set to “Bacteria”). The software automatically creates the UniProt search-query URL based on script parameters. However, this option can be overridden by either specifying a custom URL of UniProt DB search or by inputting a custom protein dataset in AA FASTA format. In the latter case, some functionalities of the pipeline, particularly those related to the taxonomic classification of protein/peptides, are not applied since necessary information is missing. On the other hand, the use of a custom protein database derived from metagenomic studies, which includes the proteins more likely to be present in the environment under investigation, can significantly enhance the effectiveness and specificity of the algorithm in the prediction of site-specific target peptides. Moreover, the copy numbers (i.e. coverage) of each protein/peptide sequence in the nucleotide FASTA file can be specified (more information is available in the GitHub README file or after invoking help message of PepMANDIS), which enables this parameter to be used as a proxy for the identification of target peptides with tentatively higher abundance in the real environment. As an advanced option, the user can also supply a dataset with protein entries in FASTA AA format. The peptides resulting from *in silico* digestion can be compared to peptides of homologous proteins previously reported in UniProt DB or other custom lists of proteins with the desired functionality. The entries presenting an exact match with the reference database can be selected as target peptides of high confidence in terms of biological functionality.

The user can also provide a config file containing information about the paths to supportive software tools, files and databases (i.e. Google Chrome driver, PeptideSieve executable and its accompanying file with amino acid physicochemical properties, BLASTP executable, BLAST nr database, as well as the file containing taxids for taxonomy restrictions of offline BLASTP search; Chrome and PeptideSieve employed for the prediction of peptide detectability by LC-MS/MS, as well as offline BLASTP used for evaluating functional specificity of peptides). However, a config file is not necessary as long as the user prefers to use Safari web browser and does not want to perform PeptideSieve analysis or offline blasting (see section “Calculation of peptide properties” below for further information). There are also multiple input parameters that can be modified in order to adjust the performance of the pipeline according to user’s needs. These parameters are explained after running the software with --help or -h option.

**FIGURE 3:**
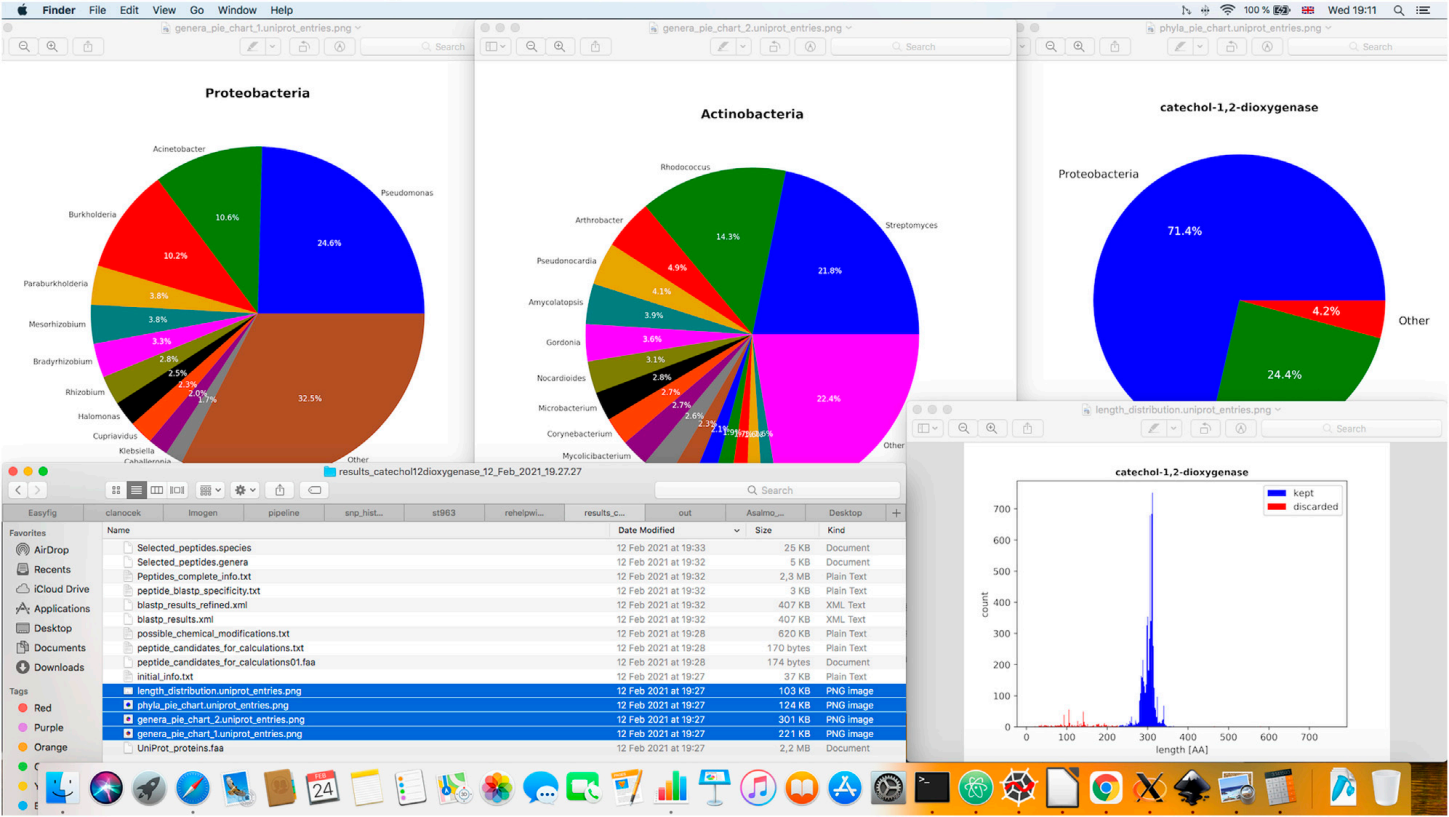
Illustration of various graphical outputs generated during PepMANDIS execution. These include three pie charts showing taxonomic composition of the protein data retrieved from UniProt DB and a histogram displaying the size distribution of the retrieved proteins (in terms of the total number of amino acid residues).

**FIGURE 4:**
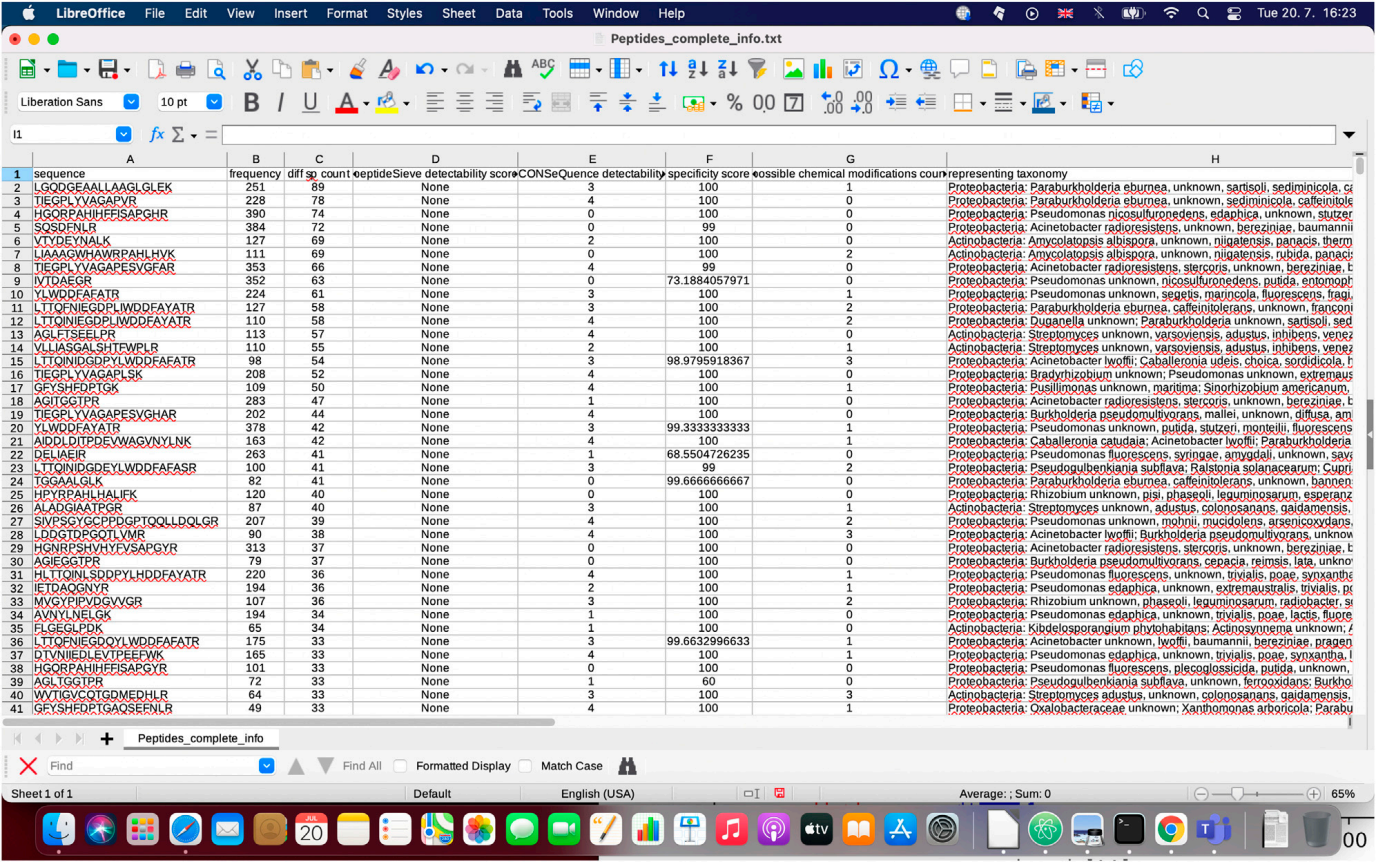
Demonstration of ‘Peptides_complete_info.txt’ file which provides comprehensive information about peptide properties calculated during the execution of PepMANDIS.

During the execution of the pipeline, several figures (see Figure 3), an XML file with the results of BLASTP search (blastp_results.xml) and various text files are outputted: a FASTA AA file of retrieved UniProt proteins (UniProt_proteins.faa), a file containing initial statistics about the input proteins (initial_info.txt), a file with the list of peptides chosen for computation of specific peptide properties (peptide_candidates_for_calculations.txt, peptide_candidates_for_calculations*.faa), a file with identified peptides prone to chemical modifications (possible_chemical_modifications.txt) and a file containing information regarding the specificity score calculations (peptide_blastp_specificity.txt). Besides the files generated in the initial stages of data processing, the ultimate resulting files include the lists of predicted best target peptides amenable to LC-MS/MS analysis (covering as many genera/species as possible, i.e. files Selected_peptides.genera, Selected_peptides.species) and a tab-delimited file that can be easily imported to MS Excel, with comprehensive information about the properties computed by PepMANDIS for all peptides of input proteins (Peptides_complete_info.txt). An example of the latter is shown in Figure 4.

### Data Processing

#### Pre-Processing of Protein Sequence Datasets

After loading input protein sequences, a pre-processing step is carried out in the following way: taxonomic information and sequence length are extracted, summary statistics for sequence lengths are calculated and the entries deviating from the median value by more than a specified percentage (by default, > 25%) are filtered out. The entries assigned with a putative function by UniProt are also removed when this option is activated. Summary statistics such as median, mean and standard deviation of protein lengths before and after refinement are calculated by Numpy (Oliphant, 2006). Taxonomy statistics (i.e. counts of proteins belonging to different phyla, genera and species) are computed as well. Afterwards, four figures are drawn employing matplotlib. pyplot functions (Hunter, 2007): a histogram of length distribution for input proteins, and pie charts showing frequencies of occurrence of input proteins among different phyla and genera. Some basic statistics on protein sequences, such as the total number of proteins entries retrieved, mean/median and standard deviation of protein sequences length, as well as the protein counts belonging to different taxonomic groups, before and after their pre-processing are also automatically exported in the form of tab-delimited text file.

#### *In silico* Protein Digestion

After pre-processing, the protein entries are proteolytically digested *in silico* with trypsin using classes from pyOpenMS library (Röst et al., 2014) and peptides having a specified length (by default, from 8 AA up to 25 AA) are stored for downstream analysis. Some key information is subsequently gathered for the resulting peptides, such as their frequency of occurrence among the different proteins, along with the total number and names of microbial species presenting a given peptide. Peptides are then sorted in descending order according to the number of species in which they were found. Finally, a list of peptides (400 by default) with the highest frequency of occurrence among microbial species are outputted into a tab-delimited text file. The specific list is subsequently used for calculations of the peptide properties in the following stage of the pipeline.

#### Integration With Metagenomic Datasets

When the protein sequences originate from custom data sources (e.g. metagenomic analysis) and the user wants to ensure that the target peptides resulting from PepMANDIS will be consistent with already verified records, two input datasets can be optionally reconciled–the primary customized dataset of proteins derived from environmental sequencing studies and annotated with the desired functionality, and a secondary dataset of verified entries derived from UniProt DB or another trusted source for the same biological function. The protein sequences of both datasets are digested *in silico* as described above and the resulting peptides with the same size are compared in a pairwise manner. The exact matches are retrieved and used in all the consequent stages of the analysis starting with the generation of the list of 400 peptides with the highest frequency of occurrence along with the highest coverage of different species.

#### Calculation of Peptide Properties

The list of top-ranking peptides is then scrutinized to determine which of them contain AA residues prone to chemical modifications. The total number and type of problematic AA are then calculated for each peptide and outputted to a tab-delimited text file. Subsequently, selenium libraries (http://www.seleniumhq.org) are used to submit via Google Chrome or Safari web browser the peptide sequences to the server running CONSeQuence tool (Eyers et al., 2011). The latter predicts the LC-MS/MS-based detectability of peptides by combining four machine learning algorithms that were trained and tested on yeast proteome data. By default, a peptide is assumed to pass the detectability filter if it is predicted to be detectable by at least one algorithm. Results are consequently retrieved and parsed by PepMANDIS. As an extra option, the user can also perform calculations with PeptideSieve (Mallick et al., 2007) (if this program is locally installed), to further evaluate the probability that a given peptide will be detectable by means of LC-MS/MS instrumentation.

The peptides included in the list are also examined for their functional specificity (i.e. whether they are encoded exclusively in proteins with desired function or not). This is done by their sequence alignment to the non-redundant (nr) protein database via BLASTP (Camacho et al., 2009) and subsequent inspection of the proteins to which they are aligned with no mismatches or gaps. BLASTP search can be conducted either online using NCBIWWW BioPython package (Cock et al., 2009) or offline if the BLAST + toolkit is installed on the computer along with the storage of nr protein database on a local disk. We recommend offline blasting since the retrieval of the results from the online search engine can take several hours or even fail entirely depending on user’s Internet connection speed and NCBI BLAST server load, whereas NCBI moves larger queries to slower computational queue. Indeed, this step is the biggest and only bottleneck of the software in terms of computational time. BLASTP search is limited to proteins occurring in selected taxonomy only (this parameter is set to “Bacteria” by default if online BLASTP search is invoked; in offline BLASTP search, taxonomy can be limited by specifying file with list of taxids). The results of BLASTP search are stored in an XML file that is then parsed by NCBIXML package from BioPython. BLASTP hits having the desired biological function are identified by comparing their description with the name of the target protein selected (user input). Since homolog proteins are frequently stored in nr database with slightly disparate names, some BLASTP hits can be erroneously regarded as not having the desired functional role. For this reason, PepMANDIS creates an exhaustive list of protein synonyms from nr database that are subsequently taken into account in the computation of functional specificity scores of peptides (i.e. the percentage of BLASTP hits with the desired biological function). A text file with all protein synonyms along with information about the specificity scores of individual peptides is outputted to enable the possibility for manual inspection of results and the detection of potential errors in specificity score calculations. In case the user suspects that some peptides are still erroneously flagged with low specificity scores and rejected from further analysis, it is possible to decrease the specificity threshold (option -s) or use option -a, which allows to define a list of peptides for which the specificity score-based filtering step will not apply. There is also the possibility of disabling the computation of peptides functional specificity (option --no-bsearch), in case the user prefers to use other tools for this purpose (e.g. Unipept).

#### Generation of Short Lists of Target Peptides

When the input dataset consists of function-specific protein sequences retrieved from UniProt DB together with taxonomy information, two lists of predicted best target peptides (50 peptides by default) are outputted. The first list includes peptides that collectively cover as many genera as possible, while the second list contains peptides comprising the highest coverage at the species level. In both cases, only the peptides that pass all filtering criteria (i.e. detectability thresholds, specificity threshold, peptide length thresholds and maximum number of allowed chemical modifications) are identified and sorted in order of decreasing contribution to genera or species coverage. For input datasets consisting of protein sequences inferred from metagenomic data without taxonomic classification, a single list of peptides with the highest frequency of occurrence among encoded proteins is generated. This relies on the premise that genes presenting multiple copies in a metagenomic analysis will be expressed at higher levels in the environmental system and the respective protein/peptides will have greater probability of detection by LC-MS/MS. Since PepMANDIS is focused on homologous genes/proteins only, this assumption is very likely to be true.

In addition to short lists, PepMANDIS also outputs a tab-delimited file containing all the peptides within the specified length thresholds derived from initial proteins (when protein/peptide sequences derived from metagenomics studies are compared against UniProt homolog entries, only the peptides having an exact match are included) together with comprehensive information, such as AA sequence, frequency of occurrence among proteins, number of different species encoding each peptide, PeptideSieve and CONSeQuence scores, functional specificity score, number of possible chemical modifications and related taxonomy. This enables the user to manually modify the short list of target peptides produced by PepMANDIS or even create a completely different list of peptides for analysis by LC-MS/MS.

### User Guide

#### Installation

PepMANDIS is distributed as a GitHub repository. Downloading its source code and installation of dependencies is straightforward and should not take more than 15 min (except for NR BLAST database download) to a command-line user of average experience. The users are encouraged to contact the authors if any troubleshooting is needed. A short version of this guide with some further examples of usage is available in README file at https://github.com/matejmedvecky/pepmandis. The very first step is to ensure that Git is installed on user’s computer. On Linux, Git can be installed through the package management tool that comes with the Linux distribution (e.g. by typing ‘sudo yum install git’ in CentOS terminal or ‘sudo apt-get install git-all’ in Ubuntu terminal). MacOS users can install it via Homebrew package manager (https://brew.sh) by typing ‘brew install git’ in MacOS terminal, while Windows users should download and run an official build, which is available at https://git-scm.com/downloads.

After Git is successfully installed, the working directory should be changed to the one where PepMANDIS is going to be installed. PepMANDIS repository with the source code, configuration files and output screenshots can then be obtained by entering ‘git clone https://github.com/matejmedvecky/pepmandis.git’ in the Terminal. PepMANDIS script is located in the ‘bin’ directory. For convenience, the path of PepMANDIS ‘bin’ directory can be added into PATH system variable. so that it can be easily executed from the command line without specifying full path to the script file.

Apart from *Python* 3, the following list of dependencies is necessary to be installed in order to successfully execute the pipeline: biopython, matplotlib, multiprocess, numpy, pyopenms and selenium. All of them can be installed via package installer for *Python* (https://pip.pypa.io/en/stable/installing/) by command ‘pip install package_name’, e.g. ‘pip install biopython’. If after installation of pyopenms, an error message comes up informing that a library cannot be loaded, ‘openssl’ library needs to be installed as well. This one can be installed via Homebrew by typing ‘brew install openssl’ in Terminal. Chrome (recommended) or Safari web browser also needs to be installed on user’s computer. In the second case, the ‘Allow Remote Automation’ option must be enabled in Safari’s Develop menu. On the other hand, Chrome users should be aware that system’s ChromeDriver must be compatible with the system’s Chrome browser version. ChromeDriver can be downloaded from https://sites.google.com/a/chromium.org/chromedriver/downloads. macOS users may experience the following exception with Chrome browser: ‘chromedriver cannot be opened because the developer cannot be verified.’ This error can be fixed by making macOS trust ChromeDriver binary by typing ‘xattr -d com.apple.quarantine /path/to/chromedriver’ in Terminal and replace ‘/path/to/’ by the absolute path of ChromeDriver (most of the times this is located in ‘/Applications’ directory).

Since NCBI generally moves large BLAST queries to slow queues, the usage of offline BLASTing option in PepMANDIS is highly recommended. This option requires to set up BLAST non-redundant database along with BLAST + software locally. For the installation of BLAST + command line applications, the users can refer to https://www.ncbi.nlm.nih.gov/books/NBK279690/. Non-redundant (NR) BLAST database can be downloaded from the FTP server https://ftp.ncbi.nlm.nih.gov/blast/db/. In order to limit offline BLASTP search by taxonomy, a file with taxonomy IDs (taxids file) has to be provided by the user (see https://www.ncbi.nlm.nih.gov/books/NBK546209/#cookbook.Limiting_a_BLAST_search_with_a). A file ‘bacterial.ids’ containing all taxids available for bacteria can be found in ‘config’ directory of PepMANDIS repository.

As an optional dependency, PeptideSieve can be obtained from https://sourceforge.net/projects/sashimi/files/peptideSieve/. However, it should be mentioned that newer versions of macOS are not able to run PeptideSieve since LD_LIBRARY_PATH and DYLD_LIBRARY_PATH cannot be loaded due to ‘System integrity protection’. The latter should be turned off in order to enable dyld library loading.

#### Configuration File

**FIGURE 5:**
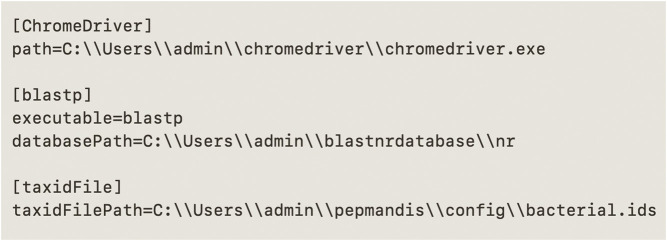
Example of a configuration file for Windows Operating System. Section headers are followed by keys and their actual values (the values represent the absolute paths to the various external files and/or executables).

Configuration file is another requirement in order to make PepMANDIS work more efficiently, if Google Chrome browser, offline BLASTP search and/or PeptideSieve tool are to be used. The structure of this file is similar to Microsoft Windows INI files. It consists of sections, starting with a [section] header on separate line, followed by keys and their values separated by ‘ = ’ character. There are four possible sections [ChromeDriver] [blastp] [PeptideSieve] and [taxidFile]. Users may include only sections that they want to use during the execution of PepMANDIS. In [ChromeDriver] section, user should provide path to CromeDriver (key is ‘path’ and value is the absolute path to ChromeDriver executable); in [blastp] section, user should provide the path to BLASTP executable (key is ‘executable’), as well as the path to NR BLAST DB (key is ‘databasePath’); in [PeptideSieve] section, there are two keys, a path to executable (key is ‘executable’) and a path to properties file (key is ‘propertiesFilePath’); in [taxidFile] section, user should specify the path to taxid file (key is ‘taxidFilePath’). A configuration file template (‘defaults.cfg’) can be found in ‘config’ directory of PepMANDIS repository. An example of a configuration file for Windows is shown in Figure 5.

#### Quick Start

On UNIX-like systems (Linux and macOS), one may need to change permissions on pepMANDIS.py file by command ‘chmod u+x pepMANDIS.py’. Several different ways of executing pepMANDIS.py along with a detailed description of its options can be found in README file at https://github.com/matejmedvecky/pepmandis. For the purposes of this user guide, it is assumed that path to pepMANDIS.py script is specified in PATH variable. The very first recommended step is to invoke help message by using the commands ‘pepMANDIS.py -h’ or ‘pepMANDIS.py --help’. It should be also noted that Windows users may need to write the python command before the actual script name (i.e. ‘py pepMANDIS.py -h’). A help message with a brief description of the software along with an explanation of all possible options should appear in the Terminal.

Generally, the command for PepMANDIS execution should be written in the form ‘pepMANDIS.py -m “desired_molecule_name” [arguments]’. Users can familiarize with PepMANDIS command line arguments and its output by running quick test instances. For example, in order to gather all catechol-1,2-dioxygenase protein entries from UniProt DB, compute basic statistics and escape running further time-consuming steps of the pipeline, the command ‘pepMANDIS.py -m “catechol-1,2-dioxygenase” --stats-only’ should be executed. To familiarize with all types of output files, one may want to run the full pipeline quickly by setting the argument -n (number of peptides with the highest species-coverage that are subjected to properties calculations) to a low number (e.g. 25), such as ‘pepMANDIS.py -m “catechol-1,2-dioxygenase” --offline-blastp -t 8 -n 25’. In the same command, offline BLASTing using eight threads has been activated by adding ‘--offline-blastp -t 8’ argument.

#### Examples of Advanced Usage

During PepMANDIS execution, it is likely that a warning message, such as “Caution: Following list of selected peptides is missing specificity scores” may show up. This may occur when the -n parameter is set to very low values. In this case, users may have to either increase -n parameter (e.g. set it to 1,000) or they can store the list of peptides with missing specificity scores into a FASTA AA file and re-run the analysis using the following command ‘pepMANDIS.py -m “catechol-1,2-dioxygenase” --no-usearch --offline-blastp -t 8 -i list_of_peptides.faa’. The argument ‘--no-usearch’ will deactivate gathering proteins from UniProt DB, while the ‘-i’ argument specifies that peptides from an input FASTA AA file will be used. Users may then manually combine the outputs from both analyses.

Given the extensive flexibility of PepMANDIS, commands of increased complexity can be used in order to execute the pipeline, such as ‘pepMANDIS.py -m “catechol-1,2-dioxygenase” -u “Actinobacteria|Proteobacteria” --offline-blastp -t 12 -a “SQSDFNLR, HGQRPAHIHFFISAPGHR,LIAAAGWHAWRPAHLHVK” -cd 2’. In this specific case, the ‘-u “Actinobacteria|Proteobacteria"’ argument restricts UniProt retrieval to protein entries belonging to either *Actinobacteria* or *Proteobacteria* phyla. In addition, the ‘-a “SQSDFNLR, HGQRPAHIHFFISAPGHR,LIAAAGWHAWRPAHLHVK"’ argument ensures that filtering of peptides SQSDFNLR, HGQRPAHIHFFISAPGHR, and LIAAAGWHAWRPAHLHVK due to unfairly low specificity scores will be avoided (the problem of biased specificity scores may result for some proteins due to the inconsistent naming of their entries in NR BLAST database). Furthermore, the ‘-cd 2’ argument specifies that at least two out of the four CONSeQuence algorithms should predict a peptide as detectable in order to avoid filtering out the peptide from the final list of best-performing candidates (i.e. increase the stringency of prediction about peptides detectability).

## Results

### Algorithm Testing

To demonstrate the functionality of PepMANDIS pipeline, we present results for two different enzymes of UniProt DB that catalyze monoaromatic compound degradation, namely catechol-1,2-dioxygenase and benzoate-1,2-dioxygenase, alpha subunit. In both cases, the default settings were used. Moreover, we present results generated using the optional input mode, where metagenomic-derived proteins/peptides of a specific biological function are integrated with homolog entries of UniProt DB and the exact matches are used for shortlisting target peptides. In this case, we utilized metagenomic data obtained from a red microbial mat at the seafloor of Santorini caldera, Greece (IMG/M system accessions 3300002231, https://img.jgi.doe.gov), indicating a high abundance of microbial genes related with the degradation of monoaromatic compounds (Oulas et al., 2016). We focused on catechol-2,3-dioxygenase enzyme, which was represented by multiple homologous genes in the specific metagenome.

### Execution Time

Execution times of PepMANDIS tool are highly dependent on whether the offline or online BLASTP search option is used. Excluding the blasting stage, all remaining parts of the pipeline were completed within minutes for the tests conducted herein (ca. 1min 30 s in the case of benzoate-1,2-dioxygenase, alpha subunit and ca. 5 min in the case of catechol-1,2-dioxygenase) when using MacBook Pro 2017 (2.3 GHz Intel Core i5). Execution times of online blasting depend on NCBI BLAST server load and they are usually shorter during weekends and weekday mornings in Europe. Generally, online blasting stage lasts from 10 min up to 2 h when 400 peptides (default value) are used as queries. Due to this inconvenience, we recommend using the offline BLASTP search option, especially when large computational resources are available for the user. Due to its higher speed, the offline option helps to increase the number of peptides that can be directed to the next stage for properties calculation. For users that do not have the capacity to perform offline BLASTP search, we introduced two options. First option (-I) enables reusage of previously generated blastp.xml file which speeds up the analysis considerably when re-running the analysis with the same dataset but different pipeline parameters. Alternatively, functional specificity calculations can be skipped completely (option–no-bsearch) and user can use third-party software for this purpose.

### Catechol-1,2-Dioxygenase

A total of 6,296 entries of catechol-1,2-dioxygenase were automatically obtained from UniProt DB. The length of sequences (number of amino acid residues) ranged from 14 to 758 AA, with a median of 305 AA, an average of 288 AA and a standard deviation of 55 AA. About 9% of the entries were removed from the dataset, as their length deviated considerably from the median value (by default, >25%). These highly diverged sequences were deemed to represent incomplete or erroneous entries. After trimming, the dataset contained 5,737 catechol-1,2-dioxygenase sequences with a length ranging from 229 to 371 AA (median: 306 AA, average: 303 AA, standard deviation: 15 AA). The filtered dataset comprised entries belonging to 12 different phyla (*Proteobacteria*, *Actinobacteria*, *Firmicutes*, *Bacteroidetes*, *Candidatus Rokubacteria*, *Acidobacteria*, *Deinococcus*-*Thermus*, *Chloroflexi*, *Planctomycetes*, *Verrucomicrobia*, *Cyanobacteria*, *Balneolaeota*), while 16 entries were of unknown taxonomy. The majority of entries belonged to *Proteobacteria* (*n* = 4,122), the most important genus of which was *Pseudomonas* (*n* = 1,031) followed by *Acinetobacter* (*n* = 437) and *Burkholderia* (*n* = 383).

*In silico* digestion resulted in 25,153 peptides, which were sorted in descending order according to their contribution to species coverage, and the properties were calculated for the top 400. The peptide LGQDGEAALLAAGLGLEK was identified as having the highest coverage at the species level (i.e. encoded in 73 different bacterial species), while it was present in 199 UniProt DB protein entries. More importantly, it passed all threshold criteria and thus represented an excellent peptide for targeted metaproteomic studies. Similarly, the most frequently encountered peptide was SQSDFNLR, found in 357 different protein entries belonging to 68 different bacterial species. However, SQSDFNLR was not included in the final short-lists as it did not pass CONSeQuence filter (i.e. low probability of being detected by LC-MS/MS). Furthermore, 76 out of the 400 peptides failed to pass CONSeQuence detectability filter, while seven peptides were below the specificity threshold and eight had more than three sites prone to chemical modifications. After discarding these 96 cases, the top 50 of the remaining peptides were added to the final short-list of target peptides. The specific peptides collectively covered 58.0 and 59.1% of genera and species registered in UniProt DB for catechol-1,2-dioxygenase.

### Benzoate-1,2-Dioxygenase, Alpha Subunit

A total of 895 sequences of benzoate-1,2-dioxygenase, alpha subunit were retrieved from UniProt DB with their length ranging from 42 to 533 AA (median: 454 AA, average: 437 AA, standard deviation: 75 AA). About 6% of them were excluded from the initial input file as being highly divergent, leading to a dataset of 842 protein sequences having a length of 363–533 (median: 455 AA, average: 455 AA, standard deviation: 14 AA). This refined dataset included three different phyla (*Proteobacteria*, *Actinobacteria*, *Bacteroidetes*), while the taxonomy of two proteins was not specified. Similarly to catechol-1,2-dioxygenase, the benzoate-1,2-dioxygenase enzyme was predominantly represented by *Proteobacteria* (*n* = 682) with *Pseudomonas* (*n* = 186) being the most important genus, followed by *Acinetobacter* (*n* = 103) and *Burkholderia* (*n* = 52).

Protein sequences were digested into 5,990 peptides. The peptide TEVTIYCIAPK exhibited the highest coverage at the species level, as it was present in 146 different bacterial species. This was also the most commonly observed peptide among individual proteins, as it recurred in 242 of them (it should be stressed that several protein entries in UniProt are attributed to uncharacterized species). Out of the 400 peptides with the highest recurrence rate in bacterial species, 174 were discarded by not meeting the criteria set for the calculated properties (62 failed to pass CONSeQuence detectability filter, 14 indicated more than three sites prone to chemical modifications and 98 did not fulfill the specificity threshold criterion). The top 50 of the remaining peptides collectively covered all different genera presenting benzoate-1,2-dioxygenase within UniProt DB, while the respective coverage at the species level was 97.2%.

With regard to specificity score calculations, some BLAST hits of peptides can be erroneously classified as irrelevant to the target protein if both their names and synonyms are completely different from those registered for all other homologous proteins in the database. Therefore, the users are strongly advised to check “peptide_blastp_specificity.txt” file, which contains information about the synonyms of the target protein and the names of all individual BLAST hits. In the case of “benzoate-1,2-dioxygenase, alpha subunit” enzyme, the majority of its tryptic peptides presented relatively low specificity scores (i.e. below 90%), because their parent proteins in BLAST database were named as “Rieske 2Fe 2S domain containing protein”, which is the short form of “Rieske 2Fe 2S domain containing protein benzoate-1,2-dioxygenase, alpha subunit”. Default specificity threshold of 90% can therefore be changed to a lower number in order to retain false negative peptides within the dataset. Lowering the specificity threshold value to 70%, the species-level coverage of the top 50 peptides increased to 100%.

### Catechol-2,3-Dioxygenase

The capability of PepMANDIS to derive target peptides from the consensus of a user-defined custom dataset (e.g. metagenomic data) and UniProt DB was tested for catechol-2,3-dioxygenase. In this operation mode, the software compares the peptides of a defined protein target that is found in a user’s sequencing study with those retrieved from UniProt DB. The exact matches from this comparison represent peptide/protein sequences that are highly likely to be expressed in the study site (e.g. as evidenced by the identification of respective exact genes in the metagenome), while their presence in UniProt DB provides an additional measure of confidence. Our custom database consisted of 43 full and partial sequences of catechol-2,3-dioxygenase derived from the metagenomic study of a red microbial mat of Santorini caldera. Despite the enormous number of catechol-2,3-dioxygenase entries retrieved from UniProt DB (17,581 protein sequences that were reduced to 11,836 after filtering highly divergent entries), only two of the resulting peptide sequences were commonly present in our custom database. The first one (IAAFLSCSNK) lacked taxonomic information as it was matched to uncultured bacteria previously reported in UniProt, while the second peptide (TIYFFDPSGNR) was ascribed to the species *Pseudomonas xanthomarina/fluorescens/monteilii/furukawaii*. The small number of exact matches suggests that the catechol-2,3-dioxygenase-like enzymes encoded by the bacteria of the microbial mat are considerably different from those stored in UniProt DB.

Given this disparity, it is advised to use metagenomic-derived sequences as the sole input dataset (i.e. deactivate cross-checking against UniProt DB), particularly when seeking target peptides for a specific family of enzymes in a largely unexplored environments. We re-analyzed our metagenomic dataset after setting parameter “−l” (length variation coefficient) of PepMANDIS to its maximum value (i.e. 100%). This modification was necessary to ensure that none of the proteins would be discarded due to protein size constraints. *In silico* digestion of the 43 sequences resulted in 185 unique peptides. Among them, eight peptides contained an unknown AA residue (i.e. the unspecified AA symbol “X”) within their sequence, 24 failed to pass CONSeQuence filter (among them also the most frequently occurring peptide LWAAWMHR that was present in four different protein entries), three peptides had more than three sites prone to chemical modifications and three did not pass specificity threshold criterion. After the application of all filtering options, there were still >100 remaining peptides for the development of LC-MS/MS-based targeted proteomic assays for the investigation of catechol-2,3-dioxygenase in microbial mat samples. Among them, 34 peptides possessed 100% specificity, contained zero AA with propensity to chemical modifications and they were predicted to be LC-MS/MS-detectable by at least one CONSeQuence algorithm, making them ideal candidates for targeted metaproteomic studies.

### Limitations

When using online BLASTP search option, long execution times can be encountered depending on NCBI server load and the user’s Internet connection speed. Sometimes, when the NCBI server load is heavy, results might not be obtained at all and the user should lower -n parameter (number of peptides to be involved in properties calculations) in order to acquire the results. Another possible limitation is related to the procedure applied for the calculation of specificity scores, which is practically based on protein names and the extensive synonyms stored in BLAST non-redundant database. The user should always check “peptide_blastp_specificity.txt” file that contains information regarding the specificity score calculations and of particular peptides with low specificity scores. Users should adjust the -s parameter (functional specificity threshold) to an appropriate value, apply optional parameter -a (i.e. define a list of peptides to be excluded from the specificity score-based filtering step), or disable functional specificity calculation completely (option --no-bsearch) and use another tool for these computations instead. Finally, other properties, such as elution time and SRM-transitions of the peptides are not predicted by PepMANDIS, but these are well covered by other specialized software (e.g. Skyline).

## Discussion

Until now, environmental proteomics were mainly oriented towards the global proteomic profiling of native soils and waters to help ecologists gain a better understanding about microbial diversity and its impact on ecosystem functioning. As opposed to the untargeted nature of classical metaproteomic studies, we here provide a bioinformatics tool that may open the way of detecting specific families of enzymes in mixed microbial communities and tracking the progress of specific biological processes with environmental or biotechnological interest. We believe that this alternative proteomic approach deserves further investigation and that PepMANDIS can significantly contribute towards this direction.

PepMANDIS is an automated pipeline with novel concept that interrogates UniProt or user-defined protein databases and calculates several protein/peptide properties and associated statistics to deduce a small list of the most representative, process-specific and MS-amenable peptides for a microbial enzymatic function of interest. Besides providing short lists of predictions for best target peptides, it generates multiple files and figures to give a comprehensive overview of their taxonomic status and assist the evaluation of some critical properties pertaining to LC-MS/MS-based targeted proteomic analysis. With regard to other software, Unipept is of the closest relevance as it is the leading platform for scrutinizing peptide lists from UniProt microbial proteomes or metaproteomic datasets. However, this is primarily oriented towards achieving comprehensive biodiversity and functional analysis of peptides rather than supporting the design of targeted proteomics methods. To our knowledge, pepMANDIS is the first-of-its-kind software explicitly developed to facilitate peptides selection for tailoring function-specific targeted metaproteomic assays in complex microbial systems. It introduces a novel approach specifically for the implementation of environmental targeted metaproteomics, but this can also find interesting applications in host-associated bacterial communities (e.g. gut microbiota). It is open source, cross-platform and offers multiple advanced features to analytical chemists working in this field. Moreover it is implemented as a Pythonic class that can be easily forked and customized by other users, since *Python* is among the most widely used languages in current biology.

## Data Availability

The datasets presented in this study, including source code, template configuration file, the file containing all bacterial taxids and software documentation, can be found in the online repository https://github.com/matejmedvecky/pepmandis.
